# Optical Biomarkers of Serous and Mucinous Human Ovarian Tumor Assessed with Nonlinear Optics Microscopies

**DOI:** 10.1371/journal.pone.0047007

**Published:** 2012-10-08

**Authors:** Javier Adur, Vitor B. Pelegati, Andre A. de Thomaz, Mariana O. Baratti, Diogo B. Almeida, L. A. L. A. Andrade, Fátima Bottcher-Luiz, Hernandes F. Carvalho, Carlos L. Cesar

**Affiliations:** 1 Biophotonic Group, Optics and Photonics Research Center (CEPOF), Institute of Physics “Gleb Wataghin”, State University of Campinas – UNICAMP, Campinas, São Paulo, Brazil; 2 Microscopy Laboratory Applied to Molecular and Cellular Studies, School of Bioengineering, National University of Entre Ríos, Oro Verde, Entre Ríos, Argentina; 3 Department of Pathological Anatomy, Faculty of Medical Sciences, State University of Campinas – UNICAMP, Campinas, São Paulo, Brazil; 4 Department of Obstetrics and Gynecology, Faculty of Medical Sciences, State University of Campinas – UNICAMP, Campinas, São Paulo, Brazil; 5 Department of Anatomy, Cell Biology, Physiology and Biophysics, Institute of Biology, State University of Campinas – UNICAMP, Campinas, São Paulo, Brazil; 6 INFABiC - National Institute of Science and Technology on Photonics Applied to Cell Biology, Campinas, São Paulo, Brazil; The Beatson Institute for Cancer Research, United Kingdom

## Abstract

**Background:**

Nonlinear optical (NLO) microscopy techniques have potential to improve the early detection of epithelial ovarian cancer. In this study we showed that multimodal NLO microscopies, including two-photon excitation fluorescence (TPEF), second-harmonic generation (SHG), third-harmonic generation (THG) and fluorescence lifetime imaging microscopy (FLIM) can detect morphological and metabolic changes associated with ovarian cancer progression.

**Methodology/Principal Findings:**

We obtained strong TPEF + SHG + THG signals from fixed samples stained with Hematoxylin & Eosin (H&E) and robust FLIM signal from fixed unstained samples. Particularly, we imaged 34 ovarian biopsies from different patients (median age, 49 years) including 5 normal ovarian tissue, 18 serous tumors and 11 mucinous tumors with the multimodal NLO platform developed in our laboratory. We have been able to distinguish adenomas, borderline, and adenocarcinomas specimens. Using a complete set of scoring methods we found significant differences in the content, distribution and organization of collagen fibrils in the stroma as well as in the morphology and fluorescence lifetime from epithelial ovarian cells.

**Conclusions/Significance:**

NLO microscopes provide complementary information about tissue microstructure, showing distinctive patterns for serous and mucinous ovarian tumors. The results provide a basis to interpret future NLO images of ovarian tissue and lay the foundation for future in vivo optical evaluation of premature ovarian lesions.

## Introduction

Epithelial ovarian cancers (EOC) constitute 90% of ovarian cancers and are particularly insidious in nature because they are not detected and do not show signs and symptoms of the disease until late stages. This entity is responsible for the highest mortality among gynecologic cancers [1], with an estimated number of 21,990 new cases and 15,460 deaths in 2011 in the United States [2]. Serous and mucinous are the most common types among EOCs. Serous adenocarcinomas often invade through the ovarian capsule and grow on the surface of the ovary. This type of carcinoma is usually large, often bilateral, and is composed in part of papillae lined by stratified cells of serous type. They are characterized by more extensive cellular budding and more confluent cellular growth. Mucinous adenocarcinomas, on the other hand, are composed of irregular cysts and glands lined by atypical mucinous cells, which are often stratified into four or more cell layers with basal nuclei [3]. The resistance to chemotherapy and the lack of sensitive diagnostic tools make ovarian cancer a disease difficult to treat [4]. The development of early diagnostic methods, before the growth of the tumor, is necessary to improve its treatment. To accomplish this, a better understanding of the fundamental changes in ovarian carcinogenesis is imperative. The objective of this paper is to show the advantages of Non-Linear Optical (NLO) Microscopy to observe the carcinogenetic changes of EOCs.

Several studies reported improved detection of metastatic ovarian cancer, but, unfortunately, the low resolution of these approaches has precluded evaluation of neoplasm at the cell level [5], [6]. The several techniques included in NLO microscopy provide sub-cellular resolution imaging capabilities in real time with biochemical selectivity that can be used to visualize collagen network, cell nuclei, cell metabolites in fixed, unfixed, stained or unstained samples, or in ex vivo, or even, in vivo, observations. In this paper we show that NLO microscopy including Two-Photon Excited Fluorescence (TPEF), Second/Third Harmonic Generation (SHG/THG) and Fluorescence Lifetime Imaging (FLIM) microscopies of EOC fixed stained and unstained samples provide a set of complementary important information to develop indicators of the disease stage and progression. We show that the normal and transformed ovarian epithelial/stromal interface is distinguishable in unstained and H&E-stained histology sections. Based on intrinsic optical biomarkers obtained for each NLO technique and using a complete set of scoring methods, we report quantitative differences between healthy and tumor ovarian tissue. The results provide a basis to interpret nonlinear images of ovarian tissue images and lay the foundation for future in vivo optical evaluation of premature ovarian lesions.

The fact that NLO microscopy can be performed in label free deep tissue have been the major driving force for its use in the study of in vivo or ex vivo, possibly in real time, biological process in cell biology. However, we believe that NLO microscopy is also an important tool for stained normal biopsy samples for several reasons. First, because fixed samples represent a frozen state of live samples from which it is possible to infer the behavior of the live cells. Second, because the calibration of a new technique against the gold standard is also a good reason to use fixed samples. Third, because different NLO techniques, especially when combined, can uncover information not apparent in the H&E section under normal brightfield observations. This would mean that a complete data set of biopsy samples stored in the world could be reanalyzed with these new techniques, which would help to understand cell/tissue changes due to diseases, such as cancer, in a time span of several years. The revision of the stored biopsies with NLO techniques also provides a detailed analysis of microanatomy, important to understand several diseases biology, which could be more important than its usefulness for the diagnostics. Finally, the NLO microscopy could allow the automatization of the analysis in clinical pathology with potential to enhance the productivity in this field.

The non linear optical signals (TPEF, SHG, and THG) depend on the chance to find more than one photon in time and space, which is hugely enhanced by the use of pulsed femtosecond laser and happens strongly only at the focus of the objective. TPEF and SHG are two photon optical processes that depend on the square of the incident light intensity, while THG is a three photon process that depends on the cube of incident light intensity. A signal that depends on the n^th^ power [n>1] of the intensity of the incident light is naturally confocal, because the signal strength is confined to the focal volume. SHG and THG are coherent second/third-order elastic non linear optical processes. Because two/three photons generate another photon with two/three times the energy of the incident photons, there is no energy released to the medium, meaning that no in focus cell photodamages is expected from these processes [7]. SHG microscopy is highly sensitive to the presence of biological structures with noncentrosymmetric molecular organization and has been successfully used to observe structural protein arrays, such as collagen [8], [9]. THG signal is only generated when a medium is optically heterogeneous within the focal volume scale [10], [11]. THG microscopy can reveal the presence of lipid bodies and highlights the nuclei within thick tissue.

TPEF, on the other hand, is a third-order non linear optical resonant and inelastic process where two photons excite an electron from the ground state. The emission after the excitation, however, is an incoherent optical process, which means that the TPEF signal observed is essentially the same of the one photon excitation fluorescence that became so important in cell biology after the 1950′s when scientific community learned to label specific biological structures. The main advantages of TPEF compared with usual fluorescence are deeper imaging capabilities, less photobleaching and stronger signal collected with non-descanned detectors (NDD). Another advantage not directly related to the technique, but very important, is the fact that the femtosecond light pulse used to excite the fluorescence will also generate SHG and THG signals which can be collected simultaneously. SHG/THG signals cannot be generated with the continuous wave [cw] lasers used for conventional fluorescence.

However, besides the fluorescence intensity imaging there is a whole set of information encoded in the fluorescence decay lifetime which can be detected with FLIM [12] and is strongly dependent on the chemical microenvironment around the fluorophores, such as pH, ion and oxygen concentration [13], [14], providing a chemical contrast imaging. This technique can be implemented in time or frequency domain detection. For the time domain, FLIM is based on pulsed lasers. Since nonlinear excitation inherently requires using a femtosecond or picosecond laser, time-domain FLIM is easily implemented.

Therefore the same light pulse could be used to generate the four imaging techniques, TPEF, FLIM, SHG and THG, simultaneously. SHG and THG can be detected in different detectors separated by their wavelengths with dichroic filters but fluorescence intensity and FLIM are generated by the same signal and detector. Just counting the number of emitted photons one gets the intensity while for FLIM one must map the arrival time of each photon at the detector. Therefore, because these four techniques are based on different contrast mechanisms they have been recognized as promising approaches for cancer diagnostics [1], [15].

Earlier NLO microscopy studies of ovarian cancer have been reported by several groups [16]–[18], which shows the potential of NLO microscopy for in vivo studies. In 2011, working with H&E-stained samples and combining TPEF-SHG-THG imaging, we obtained complementary information about the epithelial/stromal interface of ovary [19], [20]. The question about the relevance of FLIM mapping of chemical environment of fluorophores after the sample fixation process was answered by Conklin et al. [21]. Working with mouse models they compared fixed to unfixed live specimens and showed that the fixation process did not significantly impact the ability to obtain useful information from NADH and FAD measurements. Other reports also demonstrated that the chemical environment around the fluorophores is somehow preserved after the sample processing [22]. Our fixation process is similar to theirs which means, therefore, that we can compare metabolic states in different samples even after the fixing procedure. As far as we know there is no report of the use of TPEF-FLIM-SHG in ovarian cancer, although these combinations have been used to study other cancers [13], [23]–[25], and none, whatsoever, with the TPEF-FLIM-SHG-THG combination.

## Materials and Methods

### Human Ovarian Samples

Ovarian tissues were obtained from women treated at the Women’s University Hospital (CAISM), Campinas, SP, Brazil, between 1992 and 1999. Data on their treatment and follow up were obtained from medical records until 2010. The research protocol was approved by the ethics committee (Institutional Ethics Committee Faculty of Medical Sciences, Unicamp), N° 1437/2001. Because this is a retrospective study with paraffin tissue blocks, the ethics committee exempted informed consent. All procedures were in accordance with the Declaration of Helsinki and the ethical principles of the medical community.

Selected ovarian tumors were fixed in 10% formalin, paraffin-embedded, and sequentially cut in 4-µm thick sections. Tissue sections were de-waxed and examined either unstained (for FLIM analysis), or after H&E staining (for TPEF/SHG/THG analysis) using standard techniques. Each H&E-stained tissue section was classified by experienced pathologists, based on established histological criteria of the World Health Organization. In total, 34 ovarian specimens were acquired from different patients (median age, 49 years; range, 17–67) and classified as normal ovarian tissue (5 cases), serous tumors (18 cases) and mucinous tumors (11 cases). Clinical characteristics of these patients are described in Table S1. The biopsies with serous diagnosis included adenoma (n = 4), borderline (n = 3), and adenocarcinoma (n = 11), while the mucinous diagnosis included adenoma (n = 4), borderline (n = 3), and adenocarcinoma (n = 4).

To investigate the effect of the fixing process on the tissue, NLO images of formalin fixed human ovarian tissues were compared with those of the fresh tissues respectively. Fresh tissues (normal n = 2, serous borderline n = 3, and mucinous borderline n = 3) were observed within 12 hours after excision.

### Multimodal Imaging Setup

**Figure 1:**
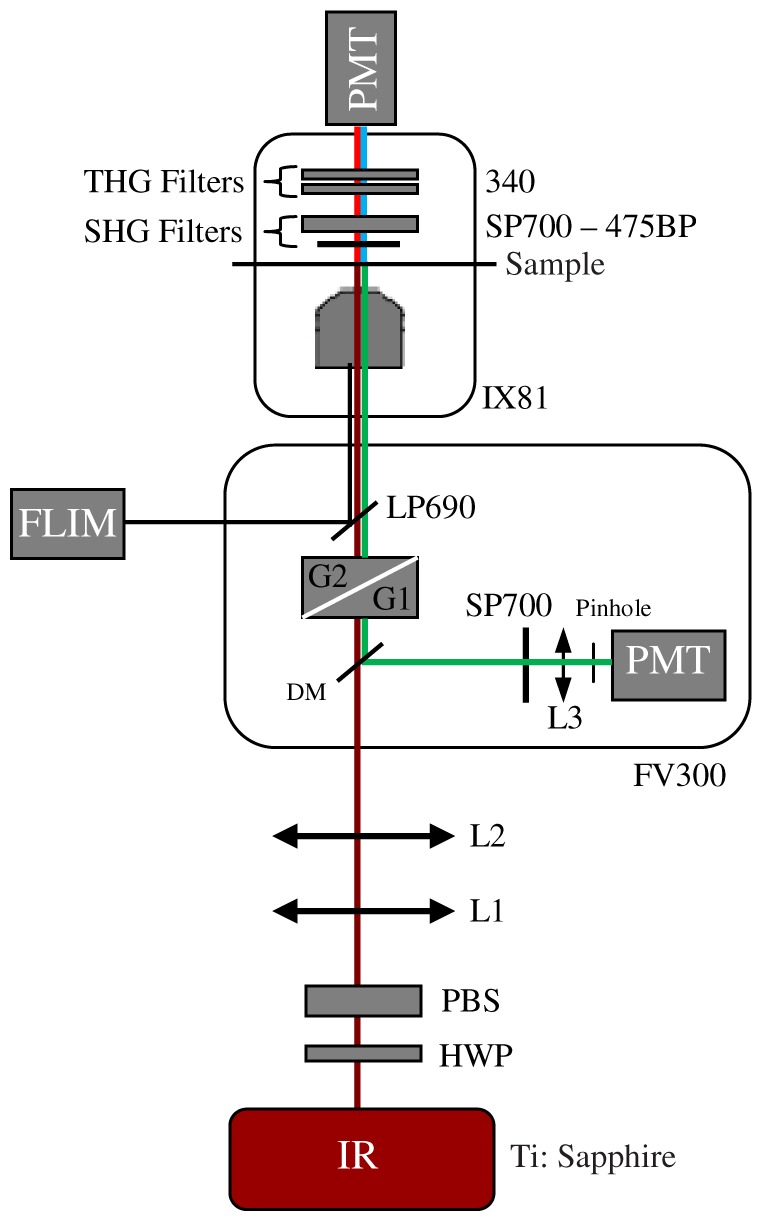
Multimodal nonlinear optical microscopy platform. Schematic representation of the multimodal platform based on an inverted microscope Olympus IX-81 and an Olympus FV300 confocal scanning head used to capture TPEF, SHG, THG, FLIM and H&E images. HWP: half wave plate, PBS: polarizing beam splitter, L1–L2: telescope lens, DM: dichroic mirror, G1–G2: galvanometer mirrors, L3: collecting lens, PMT: photomultiplier tubes, BP: bandpass filter, SP: short pass filter, LP: long pass filter. The SHG (red lines) and THG (blue lines) are collected in a transmitted light configuration. The TPEF (green lines) and FLIM (black line) are collected in back-scattering configuration.

All images were acquired with a custom multimodal NLO platform built at the Laboratory for Biophotonics of the University of Campinas, which was recently described in detail [7], [20], [26]. The system was built around an inverted microscope IX-81, equipped with an Olympus FV300 scanner (Olympus, Tokyo, Japan), as shown in figure 1. All images were acquired with a PLANAPO 40X, N.A. 1.3 oil immersion objective (Olympus, Tokyo, Japan) and were excited with a Ti:Sapphire Mai Tai HP laser of Spectra-Physics (Irvine, USA) which provides 100 fs pulses from 690–1040 nm with repetition rate of 80 MHz and an average power of 1.5 W for both wavelengths used (890 and 940 nm). SHG and THG signals were collected in the transmission mode while TPEF/FLIM was collected in the backscattering mode. The SHG signal was detected after an E700-SP (Omega Optical) short pass filter to block the excitation laser followed by a narrow (10 nm FWHM) bandpass optical filter centered at half the excitation wavelength (475BP, Bio-Rad) to reject any fluorescence. The fact that THG is in the UV region, where most of commercial optics materials and coatings do not work properly, means that it must travel the shortest optical path to the detector as possible with the minimum amount of intermediary optics. Moreover, because THG is the weakest signal it is imperative to reject all other light such as excitation laser remains, fluorescence and SHG. To accomplish this, the THG photomultiplier was placed right after the sample, and two identical band pass colored glass filter 340±30 nm (Hoya Corporation) were used to reject all other light. In this configuration transmitted SHG and THG cannot be detected simultaneously because we need to change the filters. The internal detector in the scanning unit was used only for TPEF intensity. For TPEF/FLIM a long-pass filter (LP) E-690- HP (Omega Filters) was used to reflect the TPEF/FLIM signal to a fast photon-counting PMT (Becker & Hickl, PMH-100). TCSPC card electronics (Becker & Hickl, SPC-830) allowed the direct TPEF image acquisition by a direct photocounting, which was later processed to obtain the FLIM images in the time domain.

### Image Acquisition

TPEF, SHG and THG images were excited with 20 mW (at the sample) of a 940 nm fs beam, generating a SHG at 470 nm and a THG at 313 nm. To make sure the sample did not move during the filter exchange the epi-TPEF was used as a calibration image. THG imaging was acquired simultaneously with the TPEF internal FV300 scan head photomultiplier tube (PMT) with the pinhole aperture widely open. SHG and TPEF/FLIM, on the other hand, were detected simultaneously but now using the TCSPC FLIM card. All digital processing were performed on the unprocessed images, to avoid artifacts, with ImageJ software (V1.45 NIH. USA.). FLIM data were acquired with the 890 nm excitation, which excites mainly the fluorescence of flavin adenine dinucleotide (FAD) [27], and collected for 60 s. Becker & Hickl supply two programs, one for capturing image data and a separate program to display the lifetime image. The imaging program (SPCImage Ver. 2.9, Becker & Hickl) determines the best exponential fit to the histograms at each pixel and displays lifetime data utilizing a color-mapping scheme. Wide field H&E images were acquired with a 10 MP digital camera placed on the microscope viewfinder. We acquired three images for each ovarian specimen, resulting in a total of 102 images for the quantification procedure. To perform FLIM analysis we used 3 images of the normal compared with 3 representative images of each type, serous and mucinous adenoma, borderline and adenocarcinoma, in a total of 21 images.

### Quantitative Assessment of Ovarian Epithelial/stromal Interface

All evaluations were performed in specific regions of interest (ROI) selected over cross-sectional imaging. The numbers and size of ROIs analyzed in each measurement is shown in the respective figures.

The SHG to autofluorescence aging index of dermis (SAAID) parameter was introduced for the first time to assess skin photoaging [28]. This index is a good parameter to discriminate between altered connective tissue regions such as tumor stroma [29]. To obtain this index we used the collagen-elastic tissue ratio map in the whole image of our ovarian tissue samples. TPEF and SHG images were opened in ImageJ and transformed to 8-bit image (0 to 255 gray levels) type. To separate each signal from shot noise and the dark current of the detector during subsequent analysis the data were thresholded between values of 15 and 255. The whole stroma region was selected as one ROI for each image to calculate the SAAID index using the following definition:
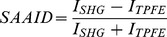
where I means the intensity of each signal, SHG/TPEF.

More analysis of collagen organization in the ovarian stroma in the ROIs presented in each figure was performed using two different image pattern analysis methods, anisotropy and texture. To evaluate anisotropy of stroma fibrils the aspect ratio of a 2-dimension intensity distribution of a Discrete Fourier Transform was used. For a complete description of this calculation see [20]. Texture features analysis, on the other hand, was based on the calculation of correlation (C), homogeneity (H), uniformity (U), and entropy (E) of the graylevel co-occurrence matrix (GLCM) of the images. The GLCM provide texture features based on gray-level statistic patterns between neighboring pixels [30]. This is done by translating the images in a parallel or perpendicular direction. We used the correlation of the image with itself, autocorrelation, translated from one to 12 pixels in the horizontal direction (perpendicular to the fibers). We then measured the distance where autocorrelation falls to 1/2, expressed in microns. A detailed description of these variables and a procedure for calculation can be found in Cichi [31] and Hall-Beyer [32], respectively.

Quantification of cytomorphological features of nuclei was performed on the THG images. At least 10 tumor cell nuclei roundness factor (Rf) morphometric variable and kurtosis of gray values densitometric variable were measured for each image analyzed [33]. To do this we standardized gray-tone scale with a range between “0” (black) and “256” (white) and then we used the “Particle Analysis-Nucleus Counter” plug-in of ImageJ. This plug-in performs a threshold automatically and presents the results in a table where each nucleus can be identified. The results table present all features previously selected in “Analyze-Set Measurements” menu (kurtosis). Area and perimeter features were selected for nuclear roundness calculations.

The SPCImage software (Becker and Hickl) was used to analyze the fluorescence lifetime decay curves, according to previous reports [34], [35]. The lifetime decay curve of each pixel was fit to a double-exponential decay model:
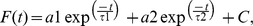
where *F*(*t*) is the fluorescence intensity at time *t* after the excitation light has ceased, τ_1_ and τ_2_ are the decay component lifetimes (τ_1_ is the short-lifetime component and τ_2_ is the long-lifetime component), *a_1_* and *a_2_* are the relative contributions of the lifetime components (i.e., a_1_+a_2_ = 100%), and *C* is a constant related to the level of background light present. The software then uses these values to build a false-color lifetime maps by assigning to each pixel in the image a color according to its lifetime value (blue and orange represents lower and higher fluorescence lifetime, respectively). The presence of two distinctly different lifetimes for free and protein-bound FAD indicates that FAD fluorescence decay curves are best fitted to a double-exponential decay model [34]. The parameter τ_i_ and *a*_i_ values were measured only in the epithelial cells and compared between different types of tumor tissues. In each image, the region of epithelium was delineated based on macroscopic appearance and histopathological correlation. The comparison between different tumor tissues was based on the 15 random selected pixels average of the average lifetime values.

### Statistical Analysis

For multi-group comparisons, one-way analysis of variance (ANOVA) with a post-hoc Tukey-Kramer test was used. We performed t-testing for two-group comparisons. The level of significance employed was significant (*) p<0.05 and very significant (**) p<0.01. Data were analyzed with SPSS 10.0 software.

## Results

**Figure 2:**
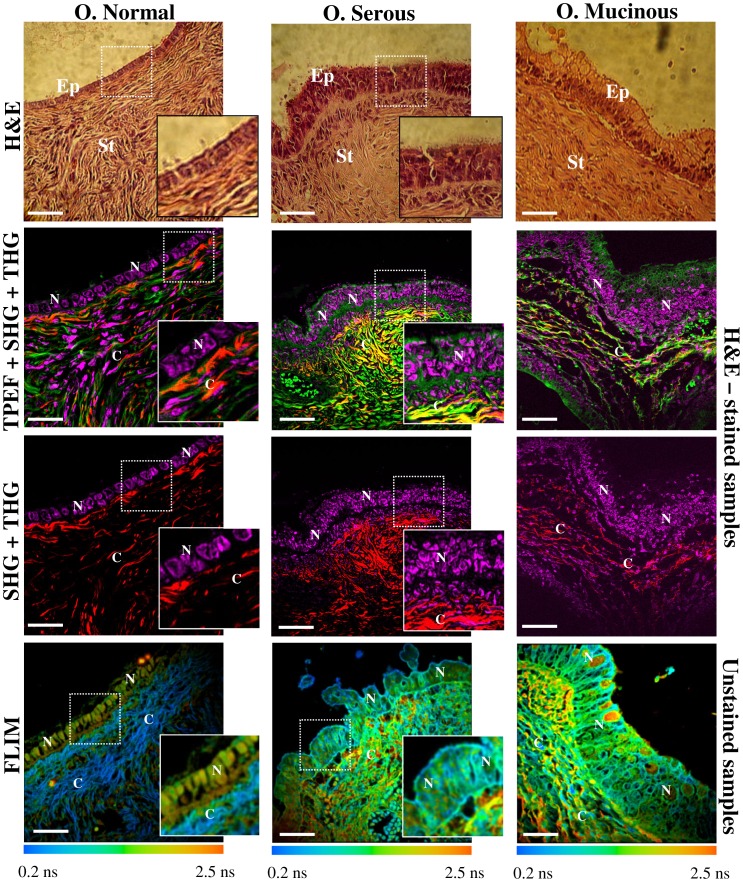
Multimodal nonlinear images of human ovary. Multicontrast cross-sectional images of human ovary. The color code for each technique are: in green TPEF; in red SHG; in magenta THG; in blue/green/yellow/orange FLIM. Column 1 represents normal ovary and column 2 and 3 represent serous and mucinous ovarian tumor types, respectively. Row 1 shows representative brightfield H&E-image. Rows 2 and 3 show different merged combinations of NLO techniques obtained from H&E-stained samples excited at 940 nm. At this wavelength TPEF signal is due to eosin fluorescence, SHG is collagen, and THG represent the nuclei. Row 4 shows FLIM images obtained from unstained samples exited at 890 nm. Blue and orange colors represent lower and higher fluorescence lifetime, respectively. SHG is also detected by the FLIM detection channel due to the choice of emission filter. SHG is an instantaneous nonlinear optical process which, therefore, appears as a very fast lifetime component (blue). In contrast, epithelial cells show lifetime values around 0.5 to 2.5 ns (yellow/orange) which match FAD endogenous fluorophor emmision. Inserts in the right of each image represents enlarged images of white square ROI where specific biomarkers could be identified (insert are not presented for mucinous tumor images). Ep: epithelium, St: stromal, N: nucleus, C: collagen fibers. Scale bars = 20 µm.

We evaluated NLO images of unstained and H&E stained samples (Figure 2). The TPEF signal is weak at the nuclear regions but strong in the regions outside the nuclei. This signal corresponded to eosin fluorescence revealing connective structures in the stroma [36]. The SHG corresponded to collagen within the connective tissue while THG highlighted the nuclei. The rich structural information revealed by each nonlinear contrast mechanism can be directly compared (row 2), providing a better understanding about the spatial organization of the tissue. For example, SHG and THG signals provide a clear contrast to distinguish the epithelial/stromal interface (row 3). Furthermore, FLIM images (row 4) show different fluorescence lifetimes between the epithelium and stromal regions and, also, a difference between different types of tumors. Representative features observed for each technique are clearly distinguishable in the enlarged images of the white square ROI of figure 2. It is worth mentioning that these differences and contrasts could be assessed automatically and digitally for quantification.

**Figure 3:**
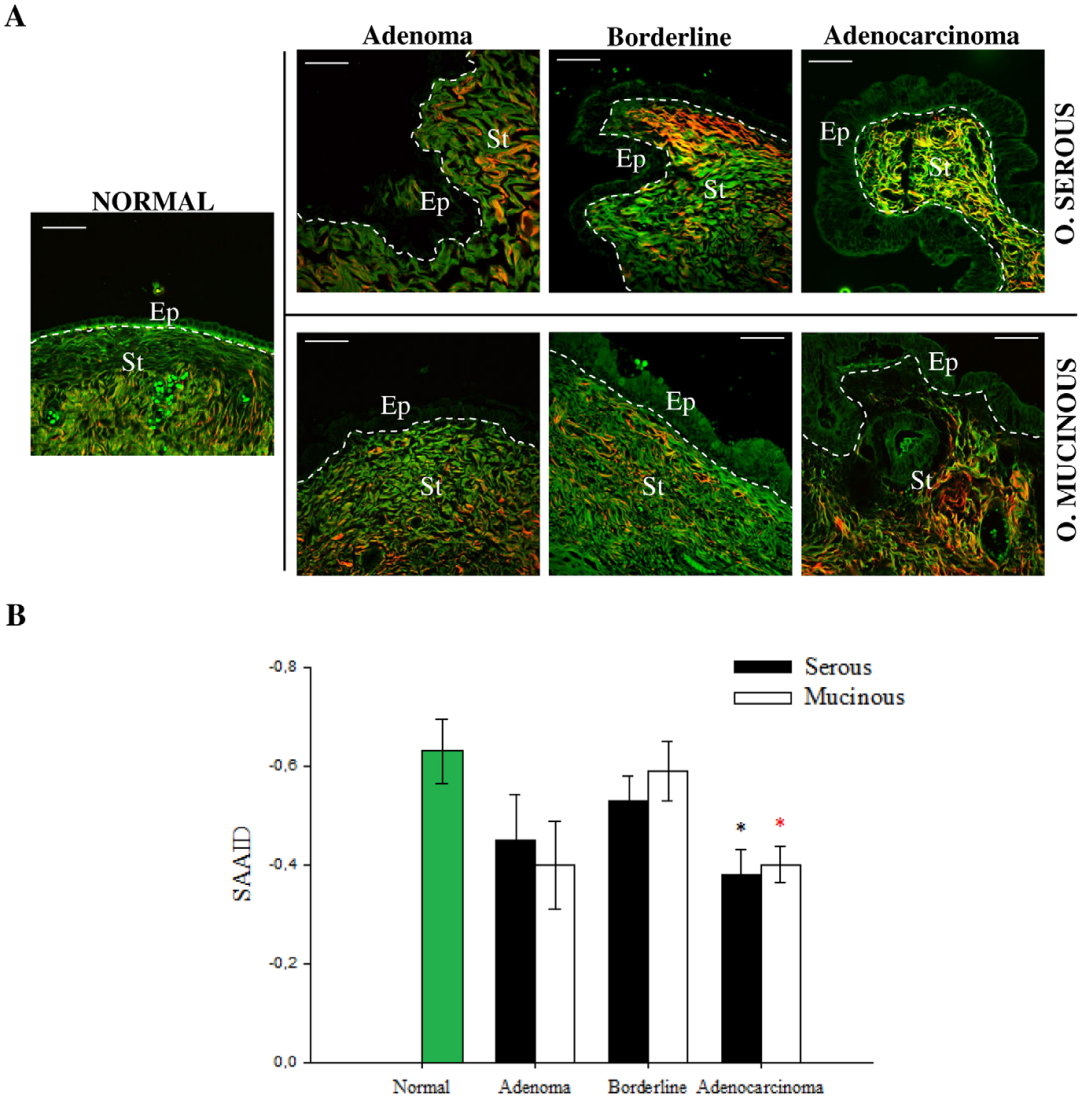
Collagen/elastin content ratio quantification in the ovarian stroma. (A) Representative merges of TPEF (green) and SHG (red) cross-sectional images of ovarian tissues obtained from H&E-stained samples. All scale bars are 20 µm. (B) SAAID index. Each bar represents the mean ±S.D. of independent measurements from stroma regions of image A. The total number of regions from which values were extracted was normal (n = 15), serous (adenoma: n = 12, borderline: n = 9, adenocarcinoma: n = 33) and mucinous (adenoma: n = 12, borderline: n = 9, adenocarcinoma: n = 12). Asterisks indicate a significant increase as compared to the non-tumor tissues (*p*<0.05, *t*-test).

We used merged TPEF+SHG signals to determine the collagen/elastin content ratio in stroma (figure 3). Collagen content increased gradually within the stroma with tumor progression, except for borderline tumors for which collagen content decreased as compared to adenomas and adenocarcinomas. The quantification of these observations is shown by the SAAID bar graph in Figure 3B. For example, the corresponding SAAID of both adenocarcinoma types presented statistically significant (*p*<0.05, *t*-test) higher values (−0.38±0.05 serous and −0.40±0.03 mucinous) compared to normal stroma (−0.63±0.06) due to the high SHG (collagen) signal and low TPEF signal in this region.

**Figure 4:**
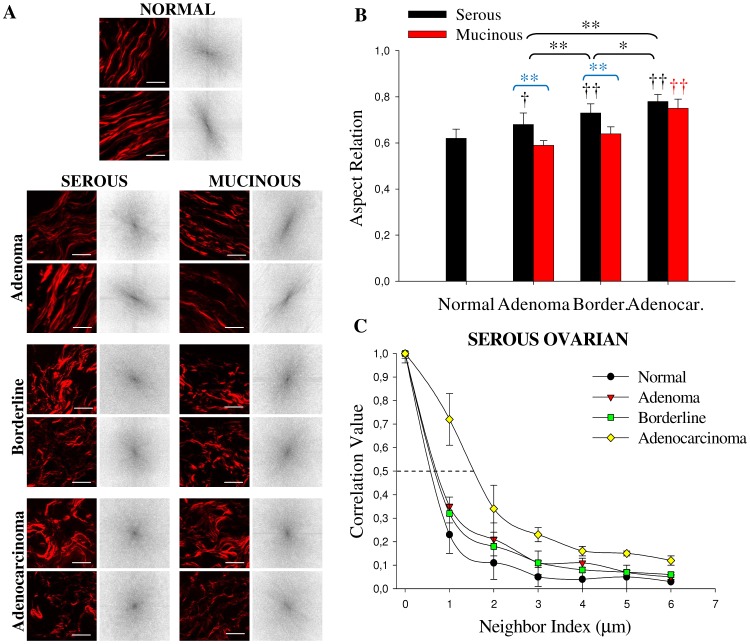
Anisotropy and texture quantification in the ovarian stromal region. (A) Representative SHG images (obtained from H&E-stained samples) of the different 150×150 pixel side yellow squared ROI in figure 4 and corresponding FFT intensity images obtained after 2D-DFT. Scale bar = 15 µm (B) Results of the aspect ratio (each bar represents the mean ±S.D. of independent measurements) of ovarian samples averaged on all ROI examined: normal (n = 45), serous (adenoma: n = 36, borderline: n = 27, adenocarcinoma: n = 99) and mucinous (adenoma: n = 36, borderline: n = 27, adenocarcinoma: n = 36). Comparisons with normal tissues are indicated with †. †,* indicates a statistically significant (p<0.05) difference and ††, ** indicates a statistically very significant (p<0.01) difference following ANOVA. (C) Correlation values in serous tumor versus pixel separation distance; the correlation for distances ranging from 1 to 18 pixels (0.35–6.0 µm) in the horizontal direction of 200 x 200 pixel ROI of interest was calculated (n = 36). Border: borderline, Adenocar: adenocarcinoma.

**Figure 5:**
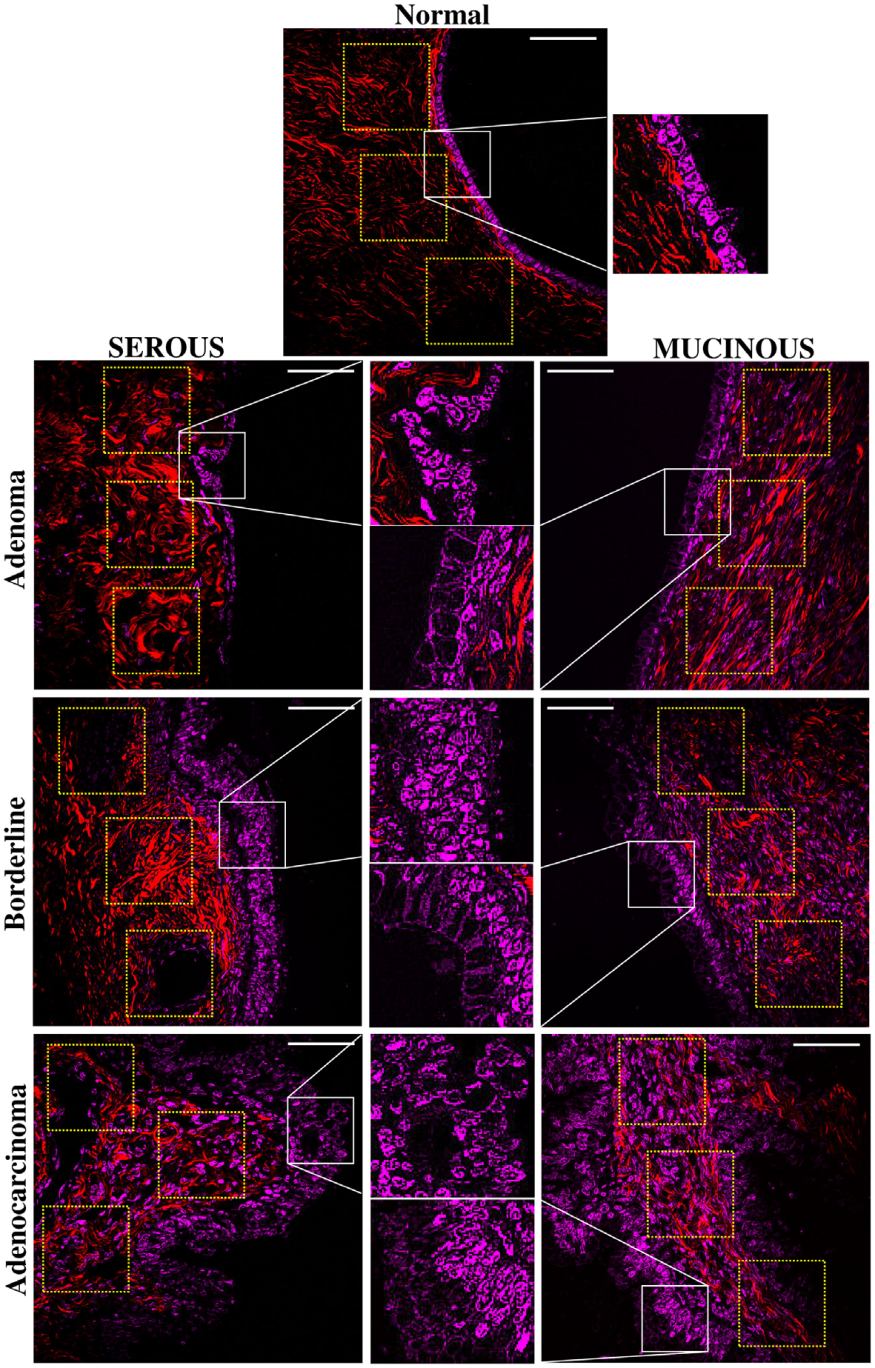
Characterization of epithelial/stromal interface using SHG+THG. Representative SHG (red) and THG (magenta) images of ovarian tissues obtained from H&E-stained samples. Yellow squares, near the epithelium, represent the selected 150×150 pixel side ROI used to perform anisotropy quantification. Insert shows more precisely the morphology of nuclei delimitated by white square. Scale bars = 20 µm.

Next, the different orientations and distribution of collagen fibers were estimated by anisotropy and texture features calculations (figure 4) performed on the SHG image of stromal region (figure 5). Anisotropy was calculated applying the Fourier transform analysis. In this case we used 3 ROI of 150×150 pixel side squared to make sure we are observing only the collagen network in the vicinity of the epithelium. Figure 4B shows the aspect ratio (AR) value averaged on all the examined samples. In serous type tumors, AR increased progressively and significantly (*p*<0.05, ANOVA) from normal (0.62±0.04) to adenoma (0.68±0.05) to borderline (0.73±0.04) and to adenocarcinoma (0.78±0.03). In contrast, in mucinous type tumors, AR showed statistically very significant differences only for adenocarcinomas (0.75±0.04) (*p*<0.01, ANOVA). Summarizing, these result confirms the fact that normal ovaries are more organized tissues as compared to adenocarcinoma. Interestingly, in the comparison between adenoma and borderline tissues of serous and mucinous tumors, AR values showed very significant differences (*p*<0.01, ANOVA), confirming that the tumor is more organized in this mucinous variant. Figure 4C shows some results of texture analysis which are complemented by data provided in Table S2. The correlation of normal fibrils fall off sharply with distance, indicating distinct, linear fibrils, whereas correlation for the fibrils in adenocarcinomas remained elevated for larger distances, implying less defined fibrillar structure. Consistent with qualitative appearances, we found that the correlation remained higher in malignant tissues with the Corr_50_, the pixel distance where the correlation dropped below 50% of the initial value, significantly greater in adenocarcinomas compared with normal ovarian (Fig. 4C; *p*<0.05, ANOVA). Similar results were found for the mucinous type tumor (data not shown). These results reflect the fact that there is a link between the epithelial carcinogenesis process and progressive loss in the fibril distribution in the stroma [37]. Normal and benign tissues show larger uniformity and homogeneity parameters values with respect to borderline and adenocarcinoma, as expected. Entropy shows the inverse tendency, meaning that normal and benign tissues are less complex tissue with respect to malignant (Table S2).

We next evaluated the differences in the surface epithelium of each type of tumor (figure 5). In non-tumor sample the cells were distributed in a single cell layer. Serous adenoma samples show tall ciliated and non-ciliated cuboidal epithelial cells with elongated nuclei, also in one single cell layer with uniform distribution. On the other hand, the serous borderline tumor and serous adenocarcinoma samples are completely different from the previous ones, showing epithelial surface with cells of different sizes distributed in multiple layers, including cellular atypia and proliferation. Mucinous tumor samples are similar to borderline/adenocarcinoma, with cells of different sizes distributed in multiple layers, but containing abundant cytoplasmic mucin and basal nuclei. The same THG details of cellular architecture were readily correlated with H-E images. The automatic quantitative evaluation of the nuclear roundness factor (Rf) and kurtosis (K) (Table S2) confirmed the visual characteristics described above. The mucinous mean Rf are significantly different from the normal although the serous are not. However the borderline/adenocarcinoma Rf standard deviations are between 5 to 10 larger than the normal/adenoma ones, indicating a more pronounced irregularity of the tumor cell nuclei.

Finally, using FLIM images, we exploited cellular FAD as an endogenous biomarker to visualize cells in epithelial ovarian. We first compared fluorescent lifetime weighted mean component (τ_m_) from epithelial cells between fixed and fresh samples at 890 nm excitation (Figure S1). Besides the expected preservation of epithelium/stroma organization after fixation the figure shows that the fluorescence intensity increased and that τ_m_ decreased in comparison with the same type fresh tissue. However the τ_m_ trends were similar in both cases, i.e., τ_m_ mucinous was larger than τ_m_ serous which was similar to τ_m_ normal. Also, cells with higher mucin content presented higher τ_m_, for both, fixed and fresh samples. This confirms the literature showing that sample fixation process did not significantly impact the fluorescence properties of FAD measurements [21]. Therefore, we decide to perform a comparison between tumors samples obtained from fixed but unstained slides.

**Figure 6:**
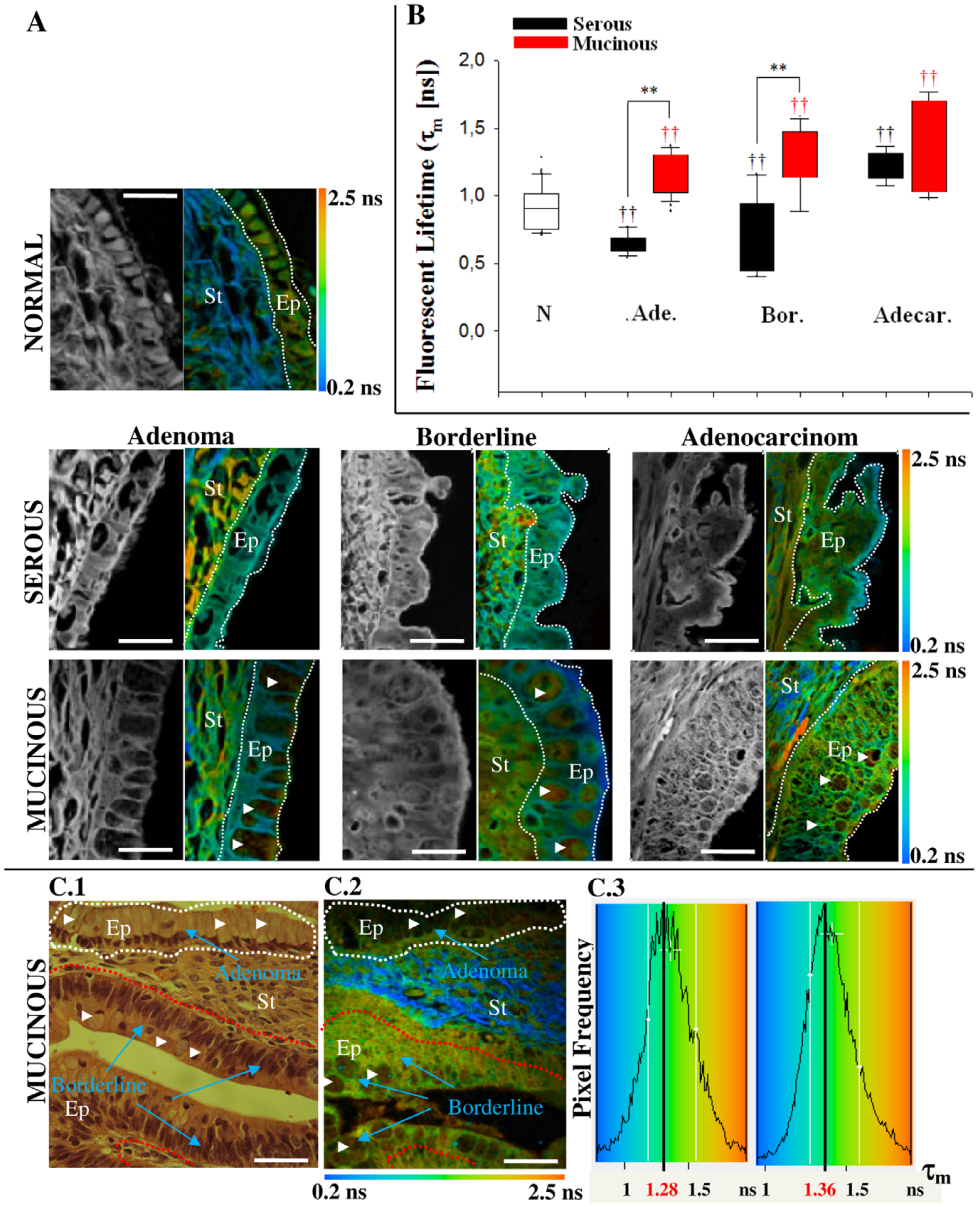
Fluorescence lifetime quantification in the ovarian epithelium. (A) Multiphoton intensity and FLIM images of endogenous fluorescence resulting from excitation at 890 nm of healthy and tumor ovarian tissues. The color map in (A) represents the weighted average of the two-term model components (τ_m_ = (*a*_1_τ_1_+*a*_2_τ_2_)/(*a*_1_+*a*_2_)) using the equation shown in the text. Scale bar = 10 µm (B) Quantitative analysis of fluorescent lifetime weighted mean component (τ_m_) calculated only in the epithelium (white dotted line). 15 pixels in different epithelial cells from each image (15×3 = 45 measurements) were used to calculate lifetime values for tumor epithelial cells. † indicates comparison with normal tissues. ††, ** indicates a statistically very significant (p<0.01) difference following ANOVA analysis. N.: normal, Ade. : adenoma, Bord.: borderline, Adecar.: Adenocarcioma. (C) C1. Digital camera image of H&E stained ovarian tumor. Adenoma to borderline transformation is indicated. C2. Color maps of the fluorescence lifetime (τ_m_), which illustrate the relatively longer lifetime values in malignant cells when compared to benign epithelium. Scale bar = 20 µm. C.3 Histogram plot (pixel frequency vs. τ_m_) of the measures for the range of lifetime values of the two ROIs drawn in (C1) reveals the shift to longer lifetimes in malignant (red line) cells compared to benign epithelium (white line). Cells with mucin are indicated with white arrowhead. Ep: epithelium, St: stromal.

Figure 6A shows the multiphoton intensity and false-color lifetime maps, while figure 6B shows the quantified τ_m_ values of epithelial cells. Epithelial cells of adenocarcinoma tissues presented a significantly (*p*<0.05, ANOVA) higher weighted mean of the fluorescent lifetime τ_m_ (serous (1.22±0.16 ns) and mucinous (1.33±0.34 ns)) as compared with the non-tumor (0.91±0.16 ns), benign tissues (serous (0.64±0.08 ns) and mucinous (1.16±0.15 ns) (Fig. 6B). This difference allows epithelial cells of malignant ovary to be easily differentiated from epithelial cells of healthy ovary, indicating a fundamentally different behavior of FAD component in these cells. It is also interesting that mucinous tumor epithelium always presented a longer lifetime value compared to serous tumor epithelium which could be used to distinguish these two types of tumors. Besides the FAD state, τ_m_ also increases with mucin content, a component not present in serous tumor. This is true independently of the fluorophore FAD state, free or bound, characterized by the short and long lifetime. Both τ_1_, bound state, and τ_2_, free state, lifetime components are longer and there was no change in the fractional contribution (*a*_1_) of each decay component (Table S2). Furthermore, τ_2_ values in mucinous tumors are higher respect to normal tissues supporting the idea that tumor cells are characterized by enhanced glycolysis [13].

In this study we could compare different stages of the mucinous tumors because we found, in the same sample, regions representing the different stages from adenoma to borderline (Fig. 6C1). This means that we have been able to observe tumor progression still in the benign early stage of the disease. We found histologically benign epithelium contiguous with tumor neoplasia, readily detected by NLO. We measured the fluorescence lifetime in the Regions of Interest ROIs traced around the adenoma (Fig. 6C1, white) and borderline (Fig. 6C1, red) epithelium such that the data could be analyzed separately. The false-color lifetime maps (Fig. 6C2) and the histogram of τ_m_ lifetime values for each ROI normalized to the peak shift to a longer lifetime (1.28 ns to 1.36 ns) in tumor cells (Fig. 6C3), which agrees with the results obtained for different samples (Fig. 6B).

## Discussion

The integration of the various microscopy techniques is one of the evolving areas in bioimaging that promises to have a strong impact on early detection of various diseases. Some reports in 2012 in human studies of bladder and liver cancer [38], [39] showed that the NLO techniques presented sensitivity, specificity and positive predictive values of 90%, 80%, 95% respectively, which means that NLO techniques represent excellent detection methodology. Our contribution cannot provide these values because many more samples and blind studies would be necessary.

The fact that NLO microscopy can see image selective specimen properties makes it easier to digitally process the images - for example, the selective imaging of the collagen fibers only or the nuclei only, opens the possibility to automatically discriminate between the different types of disease. Integration of many techniques would improve the differential diagnosis power. For example, THG sees only the nuclei pattern, an important, but insufficient, piece of information useful to discriminate different tumors. SHG would complement this with information about the extracellular matrix. In 2011 we described [20] a tumor associate collagen signature (TACS) in serous ovary where the angle of the collagen fibers with respect to epithelium was observed. These angles provide information about the invasiveness of tumors or the presence of metastasis. However, to measure the angles we needed both, SHG to see the fibers direction and the THG to delimit the epithelial surface. We also demonstrated, in this paper, that the combination of TPEF and SHG was necessary to obtain the concentrations of collagen component with respect to other stromal components. Both techniques, however, will not provide any information about the chemical environment inside cell/tissue obtained with FLIM. For example, in this work fluorescence life time of the intrinsic biologic fluorophore such as FAD was measured, presenting different values when normal vs cancer tissues were compared. The overlay of different images that can be acquired with the multimodal system would allow one to see the whole picture. Finally, for future in vivo and 3D modality studies, without the standard H&E slides for comparison, we will need all the techniques together to confirm the structures observed.

In this work, multimodal NLO microscopy successfully visualized characteristic features found in benign and malignant lesions of the human ovary. Distinctive TPEF, SHG and THG patterns were observed in the epithelial/stromal interface of the adenoma, borderline and adenocarcinoma samples. These NLO patterns were visualized both in fixed and stained samples. Previously we demonstrated the importance of the analysis of H&E-stained samples with new NLO techniques [20]. In this report we show, in agreement with other reports [36], that H&E procedure enhances the nuclei THG signal, which can be observed in stored samples. Although there are some evidences suggesting that tissue post-processing procedures may affect how intrinsic fluorescent signals respond under multiphoton excitation, previous work showed that structural proteins such as collagen and elastin are little affect by post-processing procedures [17], [36], [40]. We also did not see differences in SHG signal when comparing fixed with fresh ovarian stroma (data not show). Additionally, our and other results about TPEF obtained from fixed tissues [17], [19], [26], [36] are in line with TPEF signals obtained from fresh tissues [16], [18].

One important finding in this work was that it was possible to use anisotropy measurements to discriminate between serous adenoma from mucinous adenoma and serous borderline from mucinous borderline subtypes. Unlike ovarian serous tumors, which are relatively homogeneous in their cellular composition and degree of differentiation, mucinous tumors are frequently heterogeneous, with mixtures of benign, borderline, and malignant elements often found within the same neoplasm. They are commonly large tumors that occasionally reach massive proportions. The heterogeneity in these mucinous tumors suggests that malignant transformation is sequential and slow, progressing from cystadenoma to borderline tumor and, finally, to invasive carcinoma [41]. This slow behavior is probably reflected in a more organized stroma. We also observed that adenocarcinoma of serous and mucinous tumors have a denser distribution of collagen fibers with no defined pattern, as demonstrated by the higher collagen content, aspect ratio closer to 1, elevated correlation coefficient, lower uniformity and homogeneity values and higher entropy. THG also revealed differences between non-tumor and tumor epithelial cells. The fact that mucinous epithelium nuclei are smaller and non spherical were quantified using nuclear roundness factor and kurtosis measurements.

Recently, Williams and co-workers showed that the collagen network from human epithelial ovarian tumors was generally organized as thicker and less uniform bundles than that from normal ovaries [18]. In addition, we showed in another work, that by combining TPEF, SHG and THG imaging it is possible to detect early biomarker for the ovary epithelial tumors of the serous type [20]. Consistent with previous findings, we can say that collagen content, fiber structure, and organization are potentially key determinants of tumor cell behavior [42], [43]. Changes in the extra cellular matrix (ECM) may be a biomarker of invasion and provide insight into the factors that facilitate this process [17]. The interaction between the ECM and transformed neoplastic cells is thought to be a key element of cancer-induced angiogenesis and invasion [44]. Together with previous reports we show that multimodal NLO microscopy can detect all these endogenous biomarker.

There are several reports of endogenous fluorophores FLIM in cancer [22], [45]–[49], but this is the first work on ovarian tissues. It is known that transformed cells often experience increased glycolysis, a phenomenon known as the Warburg effect, and that the fluorescent lifetimes of NADH and FAD, and in particular the redox ratio of these two metabolites, are altered in transformed cells [50]. Moreover, it has also been reported that the fluorescence lifetime of FAD can decrease due to quenching from NAD^+^, other molecular interactions, or environmental conditions [51], [52]. Interestingly, in this work we showed that FLIM might be used to gather additional information that can be used to distinguish different types of ovarian tumors.

Similar to previous reports [13], [21], [50], we measured an increase in the fluorescence lifetime for the metabolite FAD in tumor cells. Analyzing fixed-unstained samples we detect a higher τ_m_ value in epithelium of both types of adenocarcinoma tissues relative to normal. Tryptophan, NADH and FAD are the primary endogenous fluorophores in the cells within the epithelium [53] and at 890-nm excitation the main fluorophore excited is FAD [27]. Therefore for serous adenocarcinomas tumor the state of this intracellular fluorophore is the principal responsible for the alteration of the fluorescence lifetime observed in the epithelium. In line with these results Skala and co-workers [50] recently reported an increase of the τ_1_ component of the FAD lifetime in pre-cancerous cells when compared with normal epithelium. Also in all samples evaluated, we observed higher τ_m_ values in mucinous tumor epithelium respect to serous one. We believe that these differences are due to distinct metabolic state of FAD plus an important contribution of mucin component found in cytoplasmic mucinous cells [54]. Higher fluorescence lifetime values (yellow/orange colors) in mucinous ovarian epithelium were localized fundamentally in cells with abundant mucin. This was observed in fixed (Figure 6) and fresh samples (Figure S1). However, we observed also differences in τ_m_ between mucinous tumor samples which are explained fundamentally by FAD state changes (Figure 6C).

To analyze FLIM data we considered the possibility of effects caused by formalin during fixation process (Figure S1). A recent work demonstrated that the intensity of the autofluorescence decreased significantly in fixed samples, possibly due to protein denaturation during the process of fixation and the changes in quencher concentrations [55]. However, another report, which agrees with ours, observed a larger autofluorescence in fixed samples. Probably due to the chemical or physical processes involved in the fixation that destroy other structures such as enzymes [56]. In the same work, the authors showed that fluorescence spectra of NADH and FAD show little change comparing samples stored in water or fixed in formalin, with the two fluorescence peaks present on both spectra, but that the result was different samples fixed in methanol. They pointed out that the reason why the two-photon fluorescence spectra vary differently for water and the two fixatives is due to the different underlying fixation mechanisms [57]. Formalin is a cross-linking fixative that chemically forms covalent cross-links with proteins. It has good and rapid penetration into tissue and gives good tissue and protein preservation. Tissue can therefore be preserved in formalin for a relatively long time without incurring significant changes in the microenvironments of the native fluorophores and their autofluorescence spectra. In the same line, useful NADH and FAD TPEF measurements can also be obtained from fixed tissue [21]. Our results in fixed samples are consistent with these reports and showed the same FAD fluorescence lifetime trend in both fresh and fixed samples. Furthermore, if some modification exists, we believe it should be the same for all samples processed in the same way. The fact that we detected differences in FAD component for adenoma and borderline subtype tumor in the same sample represents a strong evidence that there is a fundamental change in the lifetime for each situation.

In summary, our data demonstrated that different scoring methods extracted from images obtained with a multimodal platform of NLO microscopy techniques are useful to detect pathological changes associated with ovarian cancer progression. A challenge to pathologists is to understand which tumors are likely to progress, especially in the case of very early carcinomas. Here we present a way to detect morphological and metabolic changes associated with cancer at an early stage, with a great potential for a diagnostic tool.

## Supporting Information

Figure S1 **Fluorescence lifetime in fresh and fixed tissues.** False color maps of the fluorescence lifetime (blue and orange colors represent lower and higher fluorescence lifetime, respectively) and histogram plot (pixel frequency vs. τ_m_) in fixed unstained (left column) and fresh samples (right column) excited with 890 nm. Histograms represent the total distribution of pixels in stroma and epithelium. τ_m_ below each figure represents the fluorescent lifetime weighted mean component quantified only from pixels of epithelial cells (white dotted line). Epithelium/stroma organization was well preserved after fixation and showed increased fluorescence intensity and lower τ_m_ as compared with the same type fresh tissue. In both conditions (fixed and fresh) τ_m_ is greater in mucinous with respect to serous tumors. Cells with mucin are indicated with white arrowhead. τ_m_: fluorescent lifetime weighted mean component, n: number of biopsies analyzed, ns: nano-seconds, B: borderline, Ep: epithelium, St: stromal, Scale bar = 10 µm.
(TIF)

Table S1 **Patients clinical characteristic.**
(DOC)

Table S2 **Quantitative variables for differentiating normal, serous and mucinous ovarian samples with integrated nonlinear microscopy techniques.**
(DOC)
