# Supplementary figures and images for: Optical Biomarkers of Serous and Mucinous Human Ovarian Tumor Assessed with Nonlinear Optics Microscopies
Source: PLoS One. 2012 Oct 8;7(10):e47007. doi: 10.1371/journal.pone.0047007 (PMC3466244; doi:10.1371/journal.pone.0047007)

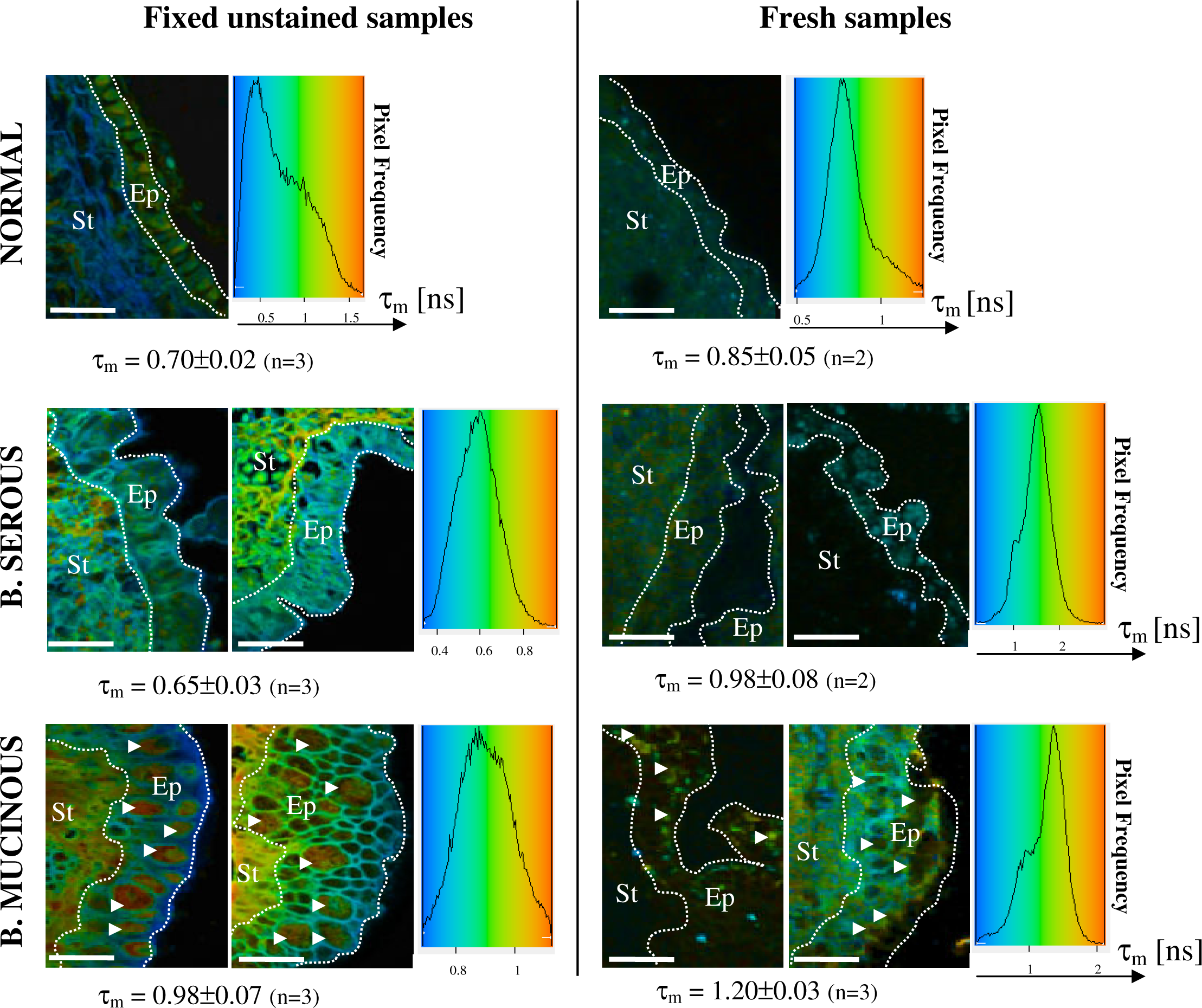

Supplement: Figure S1 — Fluorescence lifetime in fresh and fixed tissues. False color maps of the fluorescence lifetime (blue and orange colors represent lower and higher fluorescence lifetime, respectively) and histogram plot (pixel frequency vs. τm) in fixed unstained (left column) and fresh samples (right column) excited with 890 nm. Histograms represent the total distribution of pixels in stroma and epithelium. τm below each figure represents the fluorescent lifetime weighted mean component quantified only from pixels of epithelial cells (white dotted line). Epithelium/stroma organization was well preserved after fixation and showed increased fluorescence intensity and lower τm as compared with the same type fresh tissue. In both conditions (fixed and fresh) τm is greater in mucinous with respect to serous tumors. Cells with mucin are indicated with white arrowhead. τm: fluorescent lifetime weighted mean component, n: number of biopsies analyzed, ns: nano-seconds, B: borderline, Ep: epithelium, St: stromal, Scale bar = 10 µm. (TIF) [file pone.0047007.s001.tif]
