# Supplementary material for: Optical Biomarkers of Serous and Mucinous Human Ovarian Tumor Assessed with Nonlinear Optics Microscopies
Source: PLoS One. 2012 Oct 8;7(10):e47007. doi: 10.1371/journal.pone.0047007 (PMC3466244; doi:10.1371/journal.pone.0047007)
Supplement: Table S1 — Patients clinical characteristic. (DOC) [file pone.0047007.s002.doc]

Table S1: Patients clinical characteristic

| **Pat.** | **Serous Tissue** | **Age** | **Stag.** | **TG** | **Pat.** | **Mucinous Tissue** | **Age** | **Stag.** | **TG** |
| --- | --- | --- | --- | --- | --- | --- | --- | --- | --- |
| **1** | Normal | 53 |  |  | **24** | Adenoma |  |  |  |
| **2** | Normal | 46 |  |  | **25** | Adenoma |  |  |  |
| **3** | Normal | 37 |  |  | **26** | Adenoma |  |  |  |
| **4** | Normal | 33 |  |  | **27** | Adenoma |  |  |  |
| **5** | Normal | 19 |  |  | **28** | Borderline | 57 | Ia | 1 |
| **6** | Adenoma | 67 |  |  | **29** | Borderline | 49 | Ia | 2 |
| **7** | Adenoma | 57 |  |  | **30** | Adenocarcinoma | 59 | Ic | 2 |
| **8** | Adenoma | 54 |  |  | **31** | Adenocarcinoma | 53 | IIIc | 3 |
| **9** | Adenoma | 17 |  |  | **32** | Adenocarcinoma | 53 | Ia | 2 |
| **10** | Borderline | 45 | Ia | 2 | **33** | Adenocarcinoma | 43 | IIIc | 1 |
| **11** | Borderline | 39 | Ia | 1 | **34** | Adenocarcinoma | 40 | Ic | 2 |
| **12** | Borderline | 38 | IIb | 1 |  |  |  |  |  |
| **13** | Adenocarcinoma | 67 | IIa | 2 |  |  |  |  |  |
| **14** | Adenocarcinoma | 65 | IIIc | 3 |  |  |  |  |  |
| **15** | Adenocarcinoma | 64 | IIc | 3 |  |  |  |  |  |
| **16** | Adenocarcinoma | 62 | IIIc | 3 |  |  |  |  |  |
| **17** | Adenocarcinoma | 61 | IIIb | 2 |  |  |  |  |  |
| **18** | Adenocarcinoma | 59 | Ib | 1 |  |  |  |  |  |
| **19** | Adenocarcinoma | 47 | IIIa | 2 |  |  |  |  |  |
| **20** | Adenocarcinoma | 46 | IIIc | 2 |  |  |  |  |  |
| **21** | Adenocarcinoma | 43 | IIIc | 3 |  |  |  |  |  |
| **22** | Adenocarcinoma | 42 | IIIc | 3 |  |  |  |  |  |
| **23** | Adenocarcinoma | 40 | IIIc | 3 |  |  |  |  |  |

Note: Staging: according to the FIGO staging system. Abbreviations: Pat: patient, Stag: staging, TG: tumor grade.
