# Supplementary material for: Optical Biomarkers of Serous and Mucinous Human Ovarian Tumor Assessed with Nonlinear Optics Microscopies
Source: PLoS One. 2012 Oct 8;7(10):e47007. doi: 10.1371/journal.pone.0047007 (PMC3466244; doi:10.1371/journal.pone.0047007)
Supplement: Table S2 — Quantitative variables for differentiating normal, serous and mucinous ovarian samples with integrated nonlinear microscopy techniques. (DOC) [file pone.0047007.s003.doc]

Table S2: Quantitative variables for differentiating normal, serous and mucinous ovarian samples with integrated nonlinear microscopy techniques

|  |  | **Ovarian Stroma** | | | **Ovarian Epithelium** | | | | |
| --- | --- | --- | --- | --- | --- | --- | --- | --- | --- |
| **TYPE** |  | **Texture** | | | **Morphology** | | **Lifetime** | | |
|  |  | U | H | E | Rf | K | τ_1_(ns) | τ_2_(ns) | a_1_ (%) |
| **NORMAL** |  | 0.67±0.29  (n=15)^a^ | 0.77±0.14  (n=15)^a^ | 6.26±0.31  (n=15)^a^ | 0.87±0.02  (n=50)^a^ | 0.7±0.2  (n=50)^a^ | 0.53±0.15  (n=45)^a^ | 2.04±0.35  (n=45)^a^ | 75  (n=45)^a^ |
| **SEROUS** | Ade.  Bord.  Adecarc | 0.37±0.04  (n=15)^b,†^  0.30±0.03  (n=15)^b^  0.21±0.06  (n=15)^b^ | 0.60±0.18  (n=15)^a^  0.54±0.23  (n=15)^b^  0.52±0.18  (n=15)^b^ | 6.89±0.91  (n=15)^a^  7.14±0.85  (n=15)^b^  7.40±0.58  (n=15)^b^ | 0.83±0.04  (n=50)^a,†^  0.81±0.23  (n=50)^a,†^  0.72±0.31  (n=50)^b,†^ | -1.3±0.1  (n=50)^b,†^  -0.2±0.1  (n=50)^b,†^  -0.7±0.3  (n=50)^b^ | 0.46±0.07  (n=45)^a,*,†^  0.46±0.20  (n=45)^a,†^  0.85±0.15  (n=45)^b^ | 1.45±0.26  (n=45)^b,*,†^  1.34±0.48  (n=45)^b,*,†^  2.12±0.39  (n=45)^a^ | 80  (n=45)^a^  74  (n=45)^a^  70  (n=45)^a^ |
| **MUCINOUS** | Ade.  Bord.  Adecar | 0.79±0.26  (n=15)^a,*^  0.38±0.16  (n=15)^b^  0.28±0.15  (n=15)^b^ | 0.79±0.11  (n=15)^a,*^  0.66±0.13  (n=15)^a^  0.59±0.22  (n=15)^a^ | 6.24±0.39  (n=15)^a,*^  6.58±0.64  (n=15)^a^  7.07±0.75  (n=15)^b^ | 0.69±0.02  (n=50)^b,*^  0.61±0.14  (n=50)^b^  0.50±0.12  (n=50)^b^ | -1.0±0.2  (n=50)^b^  -0.8±0.5  (n=50)^b^  -0.5±0.4  (n=50)^b^ | 0.79±0.17  (n=45)^b^  0.91±0.21  (n=45)^b^  0.91±0.35  (n=45)^b^ | 2.17±0.36  (n=45)^a^  2.26±0.47  (n=45)^a^  2.15±0.55  (n=45)^a^ | 71  (n=45)^a^  70  (n=45)^a^  67  (n=45)^a^ |

Note: The average and SD of each of the variables for normal, serous and mucinous samples. The number *n* of samples or ROIs from which these parameters were extracted, indicated in parentheses, is defined as:

TEXTURE (n=15): 3 ROI (200x200) were used from each image. In total we used 5 images for each type of tissue, MORPHOLOGY (n=50): 10 nuclei were taken from each image. In total we used 5 images for each type of tissue, LIFETIME (n=45): 15 pixels (epithelial cells) were taken from each image. In total we used 3 images of each type of tissue.

Within a column, values with different superscript letters are statistically different (at least P < 0.05) and values with the same superscript letter are statistically similar (P > 0.05) compared with normal samples. * indicates a statistically significant (p < 0.05) difference comparison within serous tumor or within mucinous tumor. † indicates a statistically significant (p < 0.05) difference between serous and mucinous tumor with same diagnosis; following ANOVA analysis.

Abbreviations: Ade., adenoma, Bord., borderline, Adecarc., adenocarcinoma, U., uniformity, H., homogeneity, E., entropy, Rf., nuclear roundness factor and K., kurtosis.
